# Novel Corrosion Inhibitor for Mild Steel in HCl

**DOI:** 10.3390/ma7020662

**Published:** 2014-01-27

**Authors:** Ahmed A. Al-Amiery, Abdul Amir H. Kadhum, Abdul Hameed M. Alobaidy, Abu Bakar Mohamad, Pua Soh Hoon

**Affiliations:** 1 Department of Chemical and Process Engineering, Universiti Kebangsaan Malaysia (UKM), Bangi, Selangor 43000, Malaysia; E-Mails: amir@eng.ukm.my (A.A.H.K); drab@eng.ukm.my (A.B.M.); hoon1024psh@gmail.com (P.S.H.); 2 Environmental Research Center, University of Technology (UOT), Baghdad 10001, Iraq; E-Mails: abdulhameedalobaidy@gmail.com

**Keywords:** mild steel, thiadiazole, OCP, EIS, corrosion inhibition

## Abstract

Corrosion inhibitory effects of new synthesized compound namely 5,5’-((1Z,1’Z)-(1,4-phenylenebis(methanylylidene))bis(azanylylidene))bis(1,3,4-thiadiazole-2-thiol) (PBB) on mild steel in 1.0 M HCl was investigated at different temperatures using open circuit potential (OCP), potentiodynamic polarization (PDP) and electrochemical impedance spectroscopy (EIS). Results showed that PBB inhibited mild steel corrosion in acid solution and indicated that the inhibition efficiencies increased with the concentration of inhibitor, but decreased proportionally with temperature. Changes in impedance parameters suggested the adsorption of PBB on the mild steel surface, leading to the formation of protective films.

## 1. Introduction

**Figure 1.**
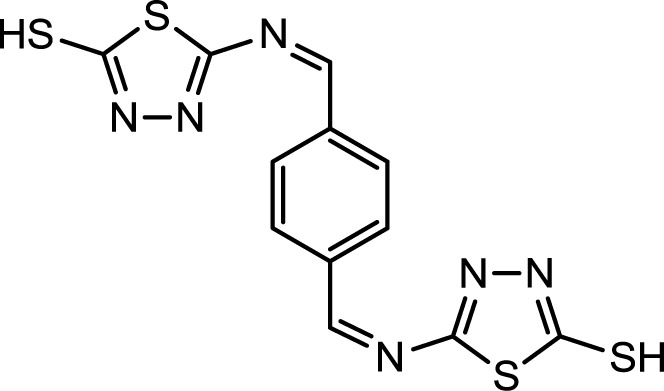
Structure of PBB.

Corrosion is a common problem for steel and directly impacts its cost and safety [1]. The majority of the well-known acid corrosion inhibitors are organic compounds that contain nitrogen, sulfur or oxygen atoms [2]. Many organic compounds have been studied to investigate their corrosion inhibition potential, e.g., the effect of organic nitrogen compounds on the corrosion behavior of iron and steel in acidic solutions; these organic nitrogen compounds are usually employed for their rapid action [3,4]. Organic inhibitors can adsorb onto the metal/solution interface via four distinct mechanisms: (a) electrostatic attraction between charged molecules and the metal; (b) interaction between uncharged electron pairs in the molecule and the metal; (c) interaction between p-electrons and the metal and (d) a combination of mechanism (a) and (c) [5]. Generally, the tendency to form stronger coordination bonds and, as a result, the inhibition efficiency increases according to the following trend: O < N < S < P [6]. The planarity (p) and lone pairs of electrons present on N, O and S atoms are important structural features that control the adsorption of these molecules onto the surface of the metal [7]. Schiff bases are effective inhibitors for the corrosion of steel in acidic media [8]. Recent studies revealed that organic compounds containing polar functions groups would be quite efficient in minimizing the effect of corrosion in addition to heterocyclic compounds containing polar groups and π-electrons [9]. As a continuation of previous studies [10–14], we focused on the synthesis of new heterocyclic compounds as novel organic corrosion inhibitors. 5,5′-((1Z,1′Z)-(1,4-phnylenebis(methanylylidene))bis(azanylylidene))bis(1,3,4-thiadiazole-2-thiol) (PBB) was synthesized in intuition to be used as a corrosion inhibitor. PBB chemical structure was elucidated and confirmed using spectroscopic techniques (IR and NMR) in addition of elemental analysis. The molecular design of the new PBB is based on the fact that 1,3,4-thiadizole containing imine, mercapto and sulfide would contribute more effectively towards inhibition of corrosion of mild steel in acid medium. Structure for the newly synthesized corrosion inhibitor is shown in Figure 1.

## 2. Results and Discussion

### 2.1. Synthesis of PBB

**Scheme 1.**
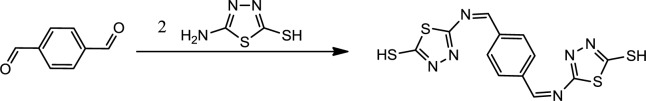
Synthesis of PBB.

The reaction sequence for synthesis of PBB is outlined in Scheme 1 was followed. The starting material was 2-amino-5-mercapto-1,3,4-thiadiazole which is commercially available or, alternatively, readily accessible through cyclization of thiosemicarbazide with carbon disulfide in presence of potassium hydroxide. Synthesis of PBB was brought about by refluxing terephthalaldehyde with 2-amino-5-mercapto-1,3,4-thiadiazole (1:2) in the presence of few drops of hydrochloric acid.

**Figure 2.**
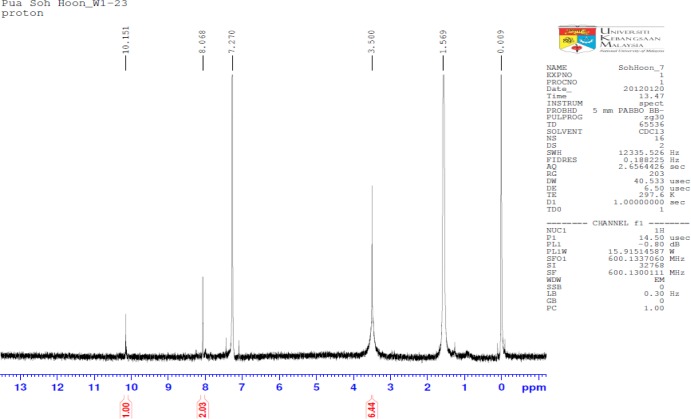
Proton NMR for PBB.

The IR spectrum is good evidence for formation of synthesized PBB, like the absence of amine group at 3300–3190 cm^−1^, and appearance new bands at 1694.7, 1608.5, 1560.04 cm^−1^. In the IR spectrum of PBB, the imines stretching frequency were observed at 1608 and 1660 cm^−1^. The high value of wave number for C=N was due to the high conjugation (resonance effect) for the substituted double bonds, whereas the aromatic carbon-carbon double bond stretching appeared at 1560 cm^−1^. Although two types of tautomers, (thione and thiol) and (amine and imine), could be expected from the PBB. The existence of the thiol form predominantly in the solid state is demonstrated by the presence of three absorption bands at 1694.7, 1608.5 and 1560.04 cm^−1^ belonging to the νC=N groups, respectively and by absence of νC=S and νNH. In the ^1^H-NMR spectrum of PBB (Figure 2), a 2H singlet was observed at 8.068 ppm due to the imines protons and a 4H singlet at 7.270 ppm due to the aromatic protons. The isolated thiol proton was observed downfield as a 2H singlet at 3.500 ppm.

### 2.2. Electrochemical Measurements

#### Open Circuit Potential (OCP)

**Figure 3.**
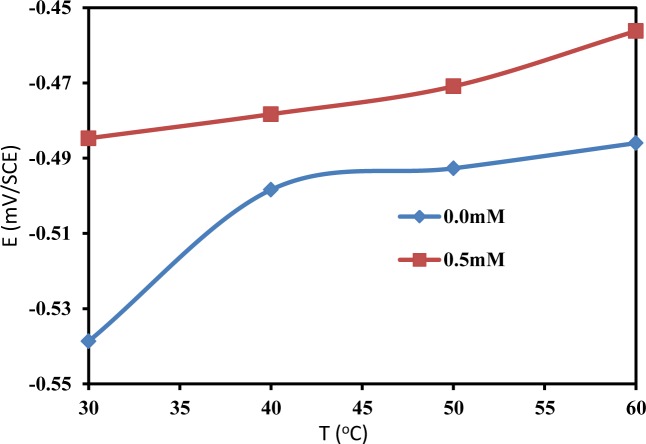
The change in OCP as a function of temperature for mild steel in 1.0 M HCl in the absence and presence of 0.5 mM PBB.

Changes in the OCP values in the presence of an inhibitor are often useful indicators of which reactions are more affected, *i.e.*, cathodic or anodic. The OCP of mild steel was monitored at different temperatures in the absence and presence of 0.5 mM PBB. Figure 3 presents the effect of the absence and presence of the PBB inhibitor on the variation of the OCP of mild steel at different temperature in 1.0 M HCl solution. It is obvious from Figure 3 that the addition of PBB to the HCl solutions shifts the OCP of the mild steel to more positive values at all of the studied temperature. This positive shift of the OCP in the presence of PBB indicates that PBB has influenced the anodic reaction of mild steel corrosion. The maximum shift in OCP was 54 mV at 30°C. This preliminary result suggests that PBB can retard the anodic reactions under open circuit conditions: the oxidation on the surface of mild steel [15,16]. In both presence and absence of PBB, the OCP values are shifted towards the positive direction with increasing temperature, reflecting the deterioration of the surface layers for the inhibitor and for the oxide, respectively.

### 2.3. Polarisation Measurements

The numerical values of the variations in corrosion current density (*i*_corr_), corrosion potential (*E*_corr_), anodic Tafel slope (β_a_), cathodic Tafel slope (β_c_) and inhibition efficiency (*IE*%) with the concentrations of inhibitor PBB at all studied temperatures are depicted in Table 1. The *IE*% is calculated as [17]:

$$IE\%=\frac{i_{\text{corr}(\text{uninh})}-i_{\text{corr}(\text{inh})}}{i_{\text{corr}(\text{uninh})}}\times 100$$ (1)

where *i*_corr(uninh)_ and *i*_corr(inh)_ are the corrosion current densities in the absence and presence of inhibitor, respectively. Close examination of Table 1 shows that an increase in temperature increases *i*_corr_, while the addition of PBB decreases the *i*_corr_ values across the temperature range. The results also indicate that the inhibition efficiencies increased with the concentration of inhibitor but decreased proportionally with temperature. Such behavior can be interpreted on the basis that the inhibitor acts by adsorbing onto the metal surface, and an increase in temperature results in desorption of some adsorbed inhibitor molecules, leading to a decrease in the inhibition efficiency [18]. In acidic solutions, the anodic reaction of corrosion is the passage of metal ions from the metal surface into the solution, and the cathodic reaction is the discharge of hydrogen ions to produce hydrogen gas or to reduce oxygen. The inhibitor may affect either the anodic or the cathodic reaction, or both [19]. Since the β_a_ and β_c_ of PBB were found to change with inhibitor concentration, this indicates that the inhibitor affected both of these reactions, however it’s more dominated on the anodic reaction based on the OCP results.

**Figure 4.**
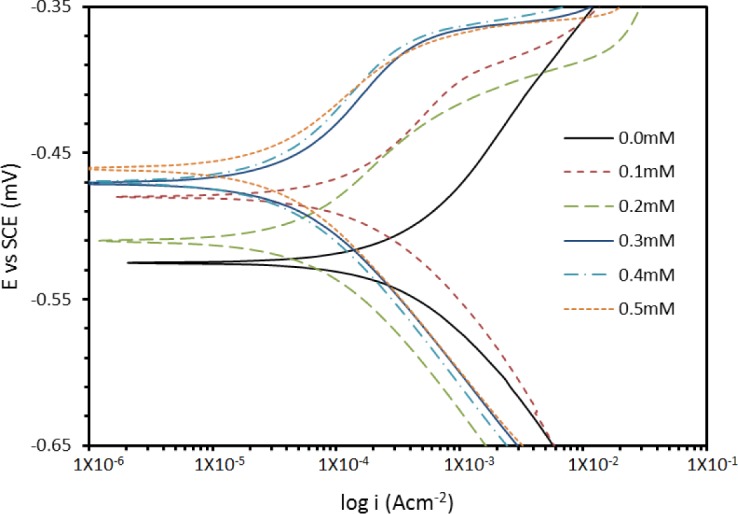
Potentiodynamic polarization curves for mild steel in 1.0 M HCl at 30°C containing various concentration of PBB.

The addition of PBB inhibitor shifts the *E*_corr_ values towards the positive; this follows the OCP trend. A compound can be classified as an anodic- or a cathodic-type inhibitor when the change in the *E*_corr_ value is larger than 85 mV [20]. Since the largest displacement exhibited by PBB was 40 mV at 30°C (Table 1), it may be concluded that this molecule should be considered as a mixed-type inhibitor. The polarization profile of mild steel in 1.0 M hydrochloric acid at 30°C in the presence and absence of PBB is shown in Figure 4. The presence of increasing amounts of PBB led to a decrease in both the cathodic and anodic current density.

Adsorption is the mechanism generally accepted to explain the inhibitory action of organic corrosion inhibitors. The adsorption of inhibitors can affect the corrosion rate in two ways: (i) by decreasing the available reaction area, the so called geometric blocking effect and (ii) by modifying the activation energy of the cathodic and/or anodic reactions occurring in the inhibitor free metal in the course of the inhibited corrosion process. It is a difficult task to determine which aspects of the inhibiting effect are connected to the geometric blocking action and which are connected to the energy effect. Theoretically, no shifts in *E*_corr_ should be observed after addition of the corrosion inhibitor if the geometric blocking effect is stronger than the energy effect [18]. The change observed in the OCP and *E*_corr_ values upon addition of PBB indicates that the energy effect is stronger, although the blocking effect cannot be ignored.

### 2.4. Electrochemical Impedance Spectroscopy (EIS) Measurements

The experimental results obtained from EIS measurements of the corrosion of mild steel in the presence and absence of inhibitor at 30, 40, 50 and 60°C are summarized in Table 2.

**Figure 5.**
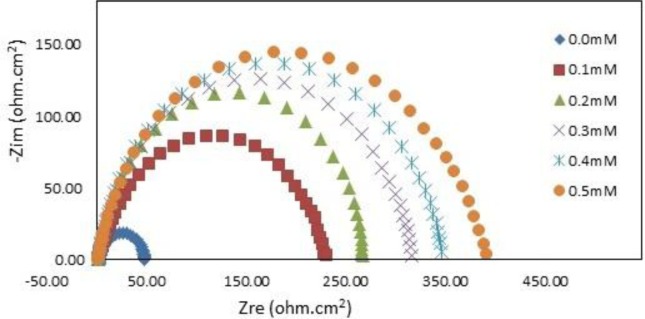
Nyquist plot for mild steel in 1.0 M HCl at 30°C containing various concentration of PBB.

The impedance spectra for mild steel in 1.0 M HCl with and without various concentrations of PBB at 30°C are presented as Nyquist plots in Figure 5.

A considerable increase in the total impedance was recorded with the addition of PBB. It can be concluded from Figure 5 that the impedance response of mild steel significantly changed after the addition of PBB in the corrosive solution. This can be attributed to an increase in substrate impedance with the increase in inhibitor concentrations. In the impedance spectra of mild steel in the absence and presence of PBB, the Nyquist plots consist of one semi-circle which attributed to the charge-transfer process. Similar behavior was noted for all of the studied temperatures. Randles equivalent circuit which consists of solution resistance (*R*_S_) in series with the parallel combination of the double-layer capacitance (*C*_dl_) and a charge-transfer resistance (*R*_ct_) was used to fit the EIS data.

$$IE\%=\frac{R_{\text{ct}(\text{inh})}-R_{\text{ct}(\text{uninh})}}{R_{\text{ct}(\text{inh})}}\times 100$$ (2)

In this equation, *R*_ct(inh)_ and *R*_ct(uninh)_ are the charge transfer resistance with and without the PBB inhibitor, respectively. Table 2 indicates that the *R*_ct_ increased and *C*_dl_ decreased with increasing PBB concentration. A large charge-transfer resistance is associated with a slowly corroding system [21]. In addition, improved inhibitor protection is associated with a decrease in the metal capacitance. The decrease in *C*_dl_, which resulted from a decrease in the local dielectric constant and/or an increase in the thickness of the electrical double layer, suggests that PBB acted via adsorption at the metal/solution interface [22]. The corrosion reaction decreased the homogeneity of the adsorbed PBB film. This was confirmed by the fact that the *C*_dl_ values increased over the temperature range. *IE*% increased as the inhibitor concentration increased, but decreased proportionally with temperature. This result follows the same general trend of the *IE*% obtained from the potentiodynamic polarization.

#### Postulated Inhibition Mechanism

**Figure 6.**
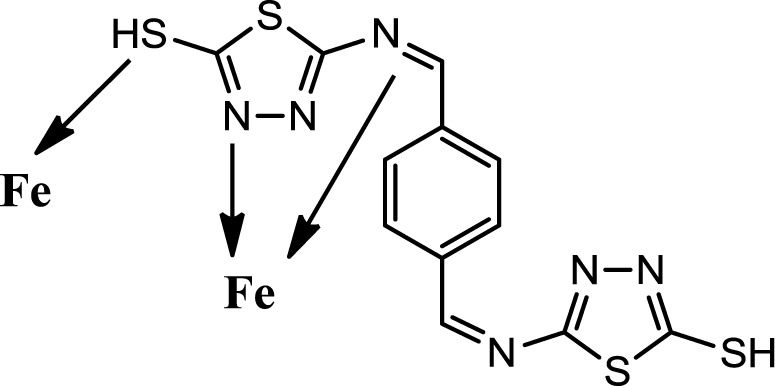
The postulated mechanism of PBB as corrosion inhibitor.

Generally organic inhibitors are adsorbed on the metal surface and prevent further dissolution of metal through blocking of either the cathodic or anodic reaction or both. Organic inhibitors, capable of forming insoluble complexes, or chelates, with metallic ions present on the surface of metal. The inhibition efficiency of our corrosion inhibitor against the corrosion of steel in 1M HCl can be explained on the basis of the number of adsorption sites, their charge density, molecular size, mode of interaction with the metal surface and the ability to form metallic complex. The π electrons and free electrons on the S and N atoms form bonds with the metal surface Figure 6.

**Scheme 2.**
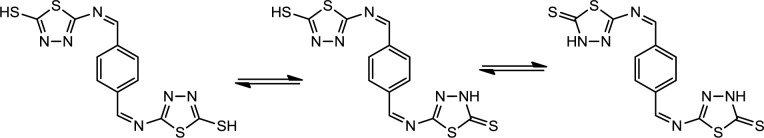
The PBB tautomerism.

The PBB can be easily protonated in the hydrochloric acid solution because the molecule is made of a planer of 1,3,4-thioziazle ring, and also contains S, and N atoms with lone pair electrons and π electrons. Coordinate covalent bond formation between electron pairs of un protonated S atom and N atoms with metal surface can take place. In addition, PBB molecules are chemically adsorbed due to the interaction of π-orbitals with metal surface. The inhibitor is very stable organic molecule it has made of a planer of 1,3,4-thioziazle ring and benzene rig in addition of double bond of azomethine group. The resonance structures of PBB give the molecule more stability Scheme 2.

**Figure 7.**
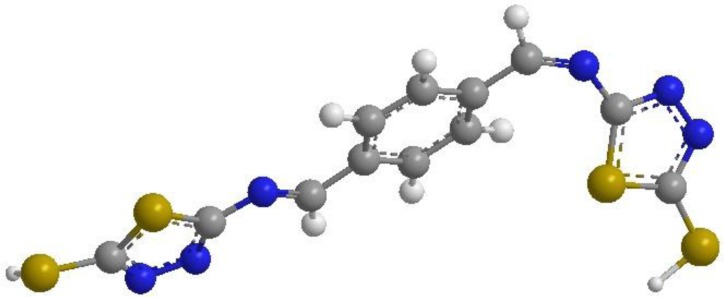
Three dimensional structure of PBB.

The inhibiting performance exhibited by the PBB may be due to the presence of protonated form of nitrogen and sulphur atoms of the PBB which makes it adsorb quickly on the mild steel surface, thus forming an insoluble stable film on the surface of the mild steel. In the present study, the value of Total Energy is 34.7675 kcal/mol, hence, showing that adsorption of PBB molecule on the surface of mild steel takes place through both physical as well as chemical processes Figure 7.

## 3. Experimental Section

### 3.1. Synthesis of Corrosion Inhibitor PBB

All chemicals used were of reagent grade (supplied by Sigma-Aldrich, Selangor, Malaysia) and used as supplied without further purifications. A solution of the 5-amino-2-mercapto-1,3,4-thiadiazole (0.4 mmol) in ethanol 25 mL was refluxed with terephthalaldehyde 0.2 mmol) for 20 h. After cooling to room temperature, a solid mass separated and recrystallized. Recrystallized from ethanol; yield 85%; ^1^H-NMR (CDCl_3_): δ 8.068, (s, 2H, N=CH), δ 7.270 (s, 4H, C–H aromatic ring) and δ 3.500 (s, 2H) for S–H; IR: 3100.0 cm^−1^ (C–H, aromatic), 1694.7 cm^−1^ (C=N, imine), 1608.5 cm^−1^ (C=N, imine), 1660.0 cm^−1^ (C=C, aromatic); Elemental Analysis: C, Anal. Calcd. for C_12_H_8_N_6_S_4_: C, 39.54%; H, 2.21%; N, 23.06%. Found: C, 38. 69%; H, 2.87%; N, 22.70%. The FTIR spectra were measured using Thermo Scientific Model Nicolate 6700 Spectrophotometer (Thermo Scientific, Hemel Hempstead, UK). NMR spectra were recorded using Model AVANCE III 600 MHz spectrometer (BRUKER, Billerica, MA, USA).

### 3.2. Electrochemical Measurements

Mild steel specimens obtained from the Metal Samples Company were used as the working electrodes throughout the study. The composition (wt%) of the mild steel was: Fe, 99.21; C, 0.21; Si, 0.38; P, 0.09; S, 0.05; Al, 0.01. The specimens were cleaned according to ASTM standard G1-03 [23]. Measurements were carried out in aerated non stirred 1.0 M HCl solutions at 30, 40, 50 and 60°C with a PBB concentration range of 0.1–0.5 mM as a corrosion inhibitor. Electrochemical measurements were conducted with Gamry water-jacketed glass cell (Gamry Instruments, Warminster, PA, USA). The cell contained three electrodes, the working, counter and reference electrodes, comprised of mild steel, a graphite bar and a calomel electrode (SCE), respectively. Measurements were performed using a Gamry Instrument Potentiostat/Galvanostat/ZRA model Ref 600. DC105 and EIS300 software by Gamry were used for potentiadynamic scans and electrochemical impedance spectroscopy (EIS). The potentiodynamic current-potential curves were swept from −0.25 to +0.25 VSCE at a scan rate of 0.5 mV·s^−1^. Impedance measurements were carried out using AC signals of 5mV peak to peak amplitude at the open circuit potential in the frequency range 0.1–100 Hz. All impedance date were fitted to appropriate equivalent circuits (EC) using Gamry Echem Analyst software. Experiments for electrochemical measurements were initiated about 30 min after the working electrode was immersed in the solution to stabilise the steady state potential. All measurements were performed in triplicate, and average values are reported.

## 4. Conclusions

5,5′-((1Z,1′Z)-(1,4-phenylenebis(methanylylidene))bis(azanylylidene))bis(1,3,4-thiadiazole-2-thiol) (PBB), was successfully synthesized and characterized using various spectroscopic methods. Electrochemical measurments such as open circuit potential (OCP), electrochemical impedance spectroscopy (EIS) and potentiodynamic polarization were used to study the corrosion inhibition of mild steel in 1.0 M HCl solutions at different temperature, using PBB as an inhibitor. This compound exhibited excellent inhibition performance as a mixed-type inhibitor. In general, the acidic corrosion of mild steel was reduced upon the addition of an appropriate concentration of PBB. The inhibition efficiencies were increased with inhibitor concentration but decreased with solution temperature.

## Figures and Tables

**Table 1.** Polarisation parameters for mild steel in 1.0 M HCl with various concentrations of PBB at different temperatures.

Temperature°C	Concentration mM	β_a_ V·dec^−1^	−β_c_ V·dec^−1^	*i*_corr_ μA·cm^−2^	−*E*_corr_ mV *vs*. SCE	*IE*%
30	0.0	0.127	0.119	461.0	525	–
30	0.1	0.078	0.104	146.0	480	68.34
30	0.2	0.064	0.090	43.8	510	90.05
30	0.3	0.076	0.091	35.2	471	92.36
30	0.4	0.085	0.095	34.3	470	92.56
30	0.5	0.058	0.086	22.5	461	95.12
40	0.0	0.188	0.145	1950	481	–
40	0.5	0.064	0.090	196.1	466	89.94
50	0.0	0.226	0.168	4320	477	–
50	0.5	0.071	0.100	604.8	480	86.00
60	0.0	0.354	0.241	14000	486	–
60	0.5	0.094	0.125	2520	487	82.00

**Table 2.** Impedance data for mild steel in 1.0 M HCl for various PBB concentrations at different temperatures.

Temperature°C	Conc. mM	*R*_ct_ ohm·cm^2^	*R*_s_ ohm·cm^2^	*C*_dl_ μF·cm^−2^	*IE*%
30	0.0	46.89	1.715	512.0	–
30	0.1	234.6	1.867	379.8	80.00
30	0.2	268.9	1.567	182.9	82.56
30	0.3	313.4	1.672	182.3	85.04
30	0.4	338.7	1.644	155.1	86.16
30	0.5	378.1	1.608	145.2	87.60
40	0.0	13.22	1.127	1226	–
40	0.5	88.13	2.122	307.7	85.00
50	0.0	7.02	1.014	1284	–
50	0.5	31.02	1.310	326.9	77.37
60	0.0	3.03	1.067	1371	–
60	0.5	8.67	1.696	691.1	65.04

## References

[1] Shukla S.K., Singh A.K., Quraishi M.A. (2012). Triazines: Efficient corrosion inhibitors for mild steel in hydrochloric acid solution. Int. J. Electrochem. Sci.

[2] Prabhu R.A., Venkatesha T.V., Shanbhag A.V., Kulkarni G.M., Kalkhambkar R.G. (2008). Inhibition effects of some Schiff’s bases on the corrosion of mild steel in hydrochloric acid solution. Corros. Sci.

[3] Myint S., Daud W.R.W., Mohamad A.B., Kadhum A.A.H. (1996). Gas chromatographic determination of eugenol in ethanol extract of cloves. J. Chromatogr.

[4] Behpour M., Ghoreishi S.M., Gandomi-Niasar A., Soltani N., Salavati-Niasari M. (2009). The inhibition of mild steel corrosion in hydrochloric acid media by two Schiff base compounds. J. Mater. Sci.

[5] Shorky H., Yuasa M., Sekine I., Issa R.M., El-Baradie H.Y., Gomma G.K. (1998). Corrosion inhibition of mild steel by schiff base compounds in various aqueous solutions. Corros. Sci.

[6] Sankarap S., Apavinasam F., Pushpanaden M., Ahmed F. (1991). Piperidine, piperidones and tetrahydrothiopyrones as inhibitors for the corrosion of copper in H_2_SO_4_. Corros. Sci.

[7] Yesim K., Seda G., Asli E. (2012). Theoretical study on the relationship between the molecular structure and corrosion inhibition efficiency of long alkyl side chain acetamide and isoxazolidine derivatives. Prot. Met. Phys. Chem. Surf.

[8] Asan A., Soylu S., Kıyak T., Yıldırım F., Öztaş S.G., Ancın N., Kabasakaloğlu M. (2006). Investigation on some Schiff bases as corrosion inhibitors for mild steel. Corros. Sci.

[9] Abdel-Gaber A.M., Masoud M.S., Khalil E.A., Shehata E.E. (2009). The effect of schiff base and its cobalt complex on the acid corrosion of steel. Corros. Sci.

[10] Junaedi S., Al-Amiery A., Kadihum A., Kadhum A.H., Mohamad A. (2013). Inhibition effects of a synthesized novel 4-aminoantipyrine derivative on the corrosion of mild steel in hydrochloric acid solution together with quantum chemical studies. Int. J. Mol. Sci.

[11] Al-Amiery A.A., Abdul A.H.K., Abu B.M., Sutiana J. (2013). A novel hydrazinecarbothioamide as a potential corrosion inhibitor for mild steel in HCl. Materials.

[12] Al-Amiery A.A., Al-Bayati R.I., Saed F.M., Ali W.B., Kadhum A.H., Mohamad A.B. (2012). Novel pyranopyrazoles: Synthesis and theoretical studies. Molecules.

[13] Kadhum A.A.H., Al-Amiery A.A., Shikara M., Mohamad A., Al-Bayati R. (2012). Synthesis, structure elucidation and DFT studies of new thiadiazoles. Int. J. Phys. Sci.

[14] Junaedi S., Kadhum A.A.H., Al-Amiery A.A., Mohamad A., Takriff M. (2012). Synthesis andcharacterization of novel corrosion inhibitor derived from oleic acid: Amino 5-Oleyl-1,3,4-Thiadiazol (AOT). Int. J. Electrochem. Sci.

[15] De Souza F.S. (2009). Caffeic acid as a green corrosion inhibitor for mild steel. Corros. Sci.

[16] Hermas A.A., Morad M.S. (2008). A comparative study on the corrosion behaviour of 304 austenitic stainless steel in sulfamic and sulfuric acid solutions. Corros. Sci.

[17] Hleli S., Abdelghani A., Tlili A. (2003). Impedance spectroscopy technique for DNA hybridization. Sensors.

[18] Wang Z. (2012). The inhibition effect of bis-benzimidazole compound for mild steel in 0.5 M HCl solution. Int. J. Electrochem. Sci.

[19] Ramesh Saliyan V., Adhikari A.V. (2008). Quinolin-5-ylmethylene-3-{[8-(trifluoromethyl)quinolin-4-yl]thio}propanohydrazide as an effective inhibitor of mild steel corrosion in HCl solution. Corros. Sci.

[20] Liu F.G., Du M., Zhang J., Qiu M. (2009). Electrochemical behavior of Q235 steel in saltwater saturated with carbon dioxide based on new imidazoline derivative inhibitor. Corros. Sci.

[21] Ashassi-Sorkhabi H., Asghari E. (2008). Effect of hydrodynamic conditions on the inhibition performance of L-methionine as a green inhibitor. Electrochim. Acta.

[22] Musa A.Y., Kadhum A.A.H., Mohamad A.B., Daud A.R., Takriff M.S., Kamarudin S.K. (2009). A comparative study of the corrosion inhibition of mild steel in sulphuric acid by 4,4-dimethyloxazolidine-2-thione. Corros. Sci.

[23] (2003). ASTM G1-3 Standard Practice for Preparing, Cleaning, and Evaluating Corrosion Test Specimens.

